# Supplementary material for: iCan, Empowering Recovery: Evaluating a Patient-Centred Cancer Rehabilitation Programme across the Cancer Care Continuum
Source: Diseases. 2024 Oct 2;12(10):236. doi: 10.3390/diseases12100236 (PMC11506657; doi:10.3390/diseases12100236)
Supplement: Supplementary file 1 [file diseases-12-00236-s001.zip › diseases-3143574-supplementary.pdf]

Table S1. Typical session structure (sessions 2-10) for the iCan cancer rehabilitation programme.

Session component	Content	Approximate timings
Pre-work	Preparatory exchange with participant via telephone and/or email. Motivational interviewing and behaviour change techniques employed.	N/A
Introduction	Participants arrive and are welcomed by instructor. Includes check to ensure no changes to health, medication, or other factors which may impact upon their ability/safety to perform the exercise component.	5 minutes
Exercise	Instructor led or circuit-based exercise following session structure principles (i.e. extended warm-up, aerobic and strength-based exercise, balance exercises, stretching/flexibility, and cool-down). Exercises include functional movements that can be repeated at home with progression/regression available depending upon ability and participant condition. All exercise intensities are self-monitored using rate of perceived exertion. Aerobic activities adapted over the programme to support attainment of participant goals.	30 minutes
Education/information	Led by instructor and focuses on the topic/theme of the session. Group discussions encouraged which include individual experiences, thoughts, feelings and actions linked to management of a cancer diagnosis.	12 minutes
Mindfulness	This can be a verbal, pre-recorded audio, or video focused mindfulness session. Linked to session topic.	8 minutes
Evaluation	Asks participants to reflect on the session, to think about how the session went. Also includes takeaway tasks for participants to complete ahead of the next session.	5 minutes
Follow-up	Each session is followed up with supporting information and best practices for all aspects of the session (linked to the topic). This is typically sent via email unless an alternative is requested by the participant.	N/A

Table S2. Changes in physical activity[1] status after completion of the iCan programme.

Outcome	PRE iCan	POST iCan
Inactive	36	7
Minimally active	43	46
HEPA active	16	42

Outcome	Change
Positive Change	
Inactive to Minimally active	21
Inactive to HEPA active	8
Minimally active to HEPA active	20
Negative Change	
HEPA active to Inactive	0
HEPA active to Minimally active	2
Minimally active to Inactive	0
No Change	
Inactive to Inactive	7
Minimally active to Minimally active	23
HEPA active to HEPA active	14

[1] Activity levels classifications were based on following criteria being met: Inactive: a) criteria for Minimally Active or HEPA Active classifications are not met; Minimally Active: a) three or more days of vigorous activity of at least 20 minutes per day or 2) five or more days of moderate-intensity activity or walking of at least 30 minutes per day or 3) five or more days of any combination of walking, moderate-intensity or vigorous intensity activities achieving a minimum of at least 600 MET-min / week; HEPA Active: a) vigorous-intensity activity on at least three days achieving a minimum of at least 1500 MET-minutes / week or 2) seven or more days of any combination of walking, moderate-intensity or vigorous intensity activities achieving a minimum of at least 3000 MET-minutes / week.
